# Multiple microRNAs targeted to internal ribosome entry site against foot-and-mouth disease virus infection *in vitro* and *in vivo*

**DOI:** 10.1186/1743-422X-11-1

**Published:** 2014-01-06

**Authors:** Yanyan Chang, Yongxi Dou, Huifang Bao, Xuenong Luo, Xuerong Liu, Kebin Mu, Zaixin Liu, Xiangtao Liu, Xuepeng Cai

**Affiliations:** 1 State Key Laboratory of Veterinary Etiological Biology, Lanzhou Veterinary Research Institute, Chinese Academy of Agricultural Sciences, Lanzhou 730046, Gansu, P. R of China; 2 Research & Development Department, China Agricultural Veterinary Biological Science and Technology Co., LTD, Lanzhou 730046, Gansu, P. R. of China; 3 China Animal Disease Prevention Control Center, Beijing 100125, P. R. of China

**Keywords:** Foot-and-mouth disease virus, MicroRNA, Internal ribosome entry site, Transformed cell clones, Antiviral effect, Flow cytometry, Real-time quantitative RT-PCR

## Abstract

**Background:**

Foot-and-mouth disease virus (FMDV) causes a severe vesicular disease in domestic and wild cloven-hoofed animals. Because of the limited early protection induced by current vaccines, emergency antiviral strategies to control the rapid spread of FMD outbreaks are needed.

Here we constructed multiple microRNAs (miRNAs) targeting the internal ribosome entry site (IRES) element of FMDV and investigated the effect of IRES-specific miRNAs on FMDV replication in baby hamster kidney (BHK-21) cells and suckling mice.

**Results:**

Four IRES-specific miRNAs significantly reduced enhanced green fluorescent protein (EGFP) expression from IRES-EGFP reporter plasmids, which were used with each miRNA expression plasmid in co-transfection of BHK-21 cells. Furthermore, treatment of BHK-21 cells with Bi-miRNA (a mixture of two miRNA expression plasmids) and Dual-miRNA (a co-cistronic expression plasmid containing two miRNA hairpin structures) induced more efficient and greater inhibition of EGFP expression than did plasmids carrying single miRNA sequences.

Stably transformed BHK-21 cells and goat fibroblasts with an integrating IRES-specific Dual-miRNA were generated, and real-time quantitative RT-PCR showed that the Dual-miRNA was able to effectively inhibit the replication of FMDV (except for the Mya98 strain) in the stably transformed BHK-21 cells.

The Dual-miRNA plasmid significantly delayed the deaths of suckling mice challenged with 50× and 100× the 50% lethal dose (LD_50_) of FMDV vaccine strains of three serotypes (O, A and Asia 1), and induced partial/complete protection against the prevalent PanAsia-1 and Mya98 strains of FMDV serotype O.

**Conclusion:**

These data demonstrate that IRES-specific miRNAs can significantly inhibit FMDV infection *in vitro* and *in vivo*.

## Background

Foot-and-mouth disease is an acute, highly contagious and economically important disease that affects domestic and wild cloven-hoofed animals, such as cattle, swine, sheep and goats [1,2]. The etiological agent, foot-and-mouth disease virus (FMDV), belongs to the genus *Aphthovirus* in the family *Picornaviridae*[3]. There are seven serotypes of FMDV and multiple subtypes [4-6]. The viral genome is composed of a positive-sense, single-stranded RNA that functions as an mRNA and contains a unique open reading frame (ORF) encoding a viral polyprotein. This polyprotein is co-translationally processed, largely by virus-encoded proteases, to produce about 15 mature proteins plus many different precursors [7-9]. Initiation of FMDV RNA translation is directed by a large RNA *cis*-acting element of about 440 nucleotides (nts) termed the internal ribosome entry site (IRES) element [10,11]. This region is predicted to adopt a secondary structure that mediates RNA–protein interactions essential for ribosome recognition [12,13]. The RNA genome also has to act as the template for RNA replication [14]. During this process, the genome undergoes rapid mutation at average rates of 10^-3^ to 10^-5^ substitution per nucleotide copied, due to the lack of proofreading mechanism of the RNA-dependent RNA polymerase (RdRp) [15-17]. Thus, FMDV populations form as “clouds” of mutants, or mutant distributions, termed viral quasispecies [18-21]. FMDV evolution is strongly influenced by high mutation rates and the dynamics of viral quasispecies, and results in ever-changing targets for antiviral strategies, including vaccination [22,23].

Although the current FMD vaccines play an essential role in the control of FMD outbreaks, they fail to induce an immediate protective response. There is a “window”, a so-called immune blank period, of susceptibility to FMDV infection in vaccinated animals at 1–7 days post-immunization [24,25]. Hence, alternative emergency strategies are needed for rapid control of FMDV outbreaks. Small interference RNAs (siRNAs) have been widely studied as a means of inhibiting FMDV replication *in vitro* and *in vivo*[26-33]. However, the efficacy and specificity of this inhibition could be completely abolished by a single point mutation in the target sequence [34-37], potentially limiting the usefulness of this approach against rapidly mutating and mutated viruses such as FMDV [38].

Therefore, the use of microRNA (miRNA), rather than siRNA, may be necessary, to cause mRNA degradation in a sequence-specific manner or gene silencing in an imperfectly base-paired manner [39,40]. Here we report that multiple vector-delivered, IRES-specific miRNAs effectively and specifically silence EGFP (enhanced green fluorescent protein) expression from IRES-EGFP fusion protein reporter plasmids in BHK-21 cells and inhibit virus replication in FMDV-IRES-specific Dual-miRNA-transformed BHK-21 cells and suckling mice. Additionally, a high-efficiency Dual-miRNA targeted to the IRES element was integrated stably into the chromosomal DNA of goat fibroblasts, for the future creation of transgenic animals resistant to FMDV infection.

## Results

### IRES-specific miRNAs on plasmids silence reporter gene expression in BHK-21 cells

#### Specific silencing of EGFP expression by a single miRNA

**Figure 1:**
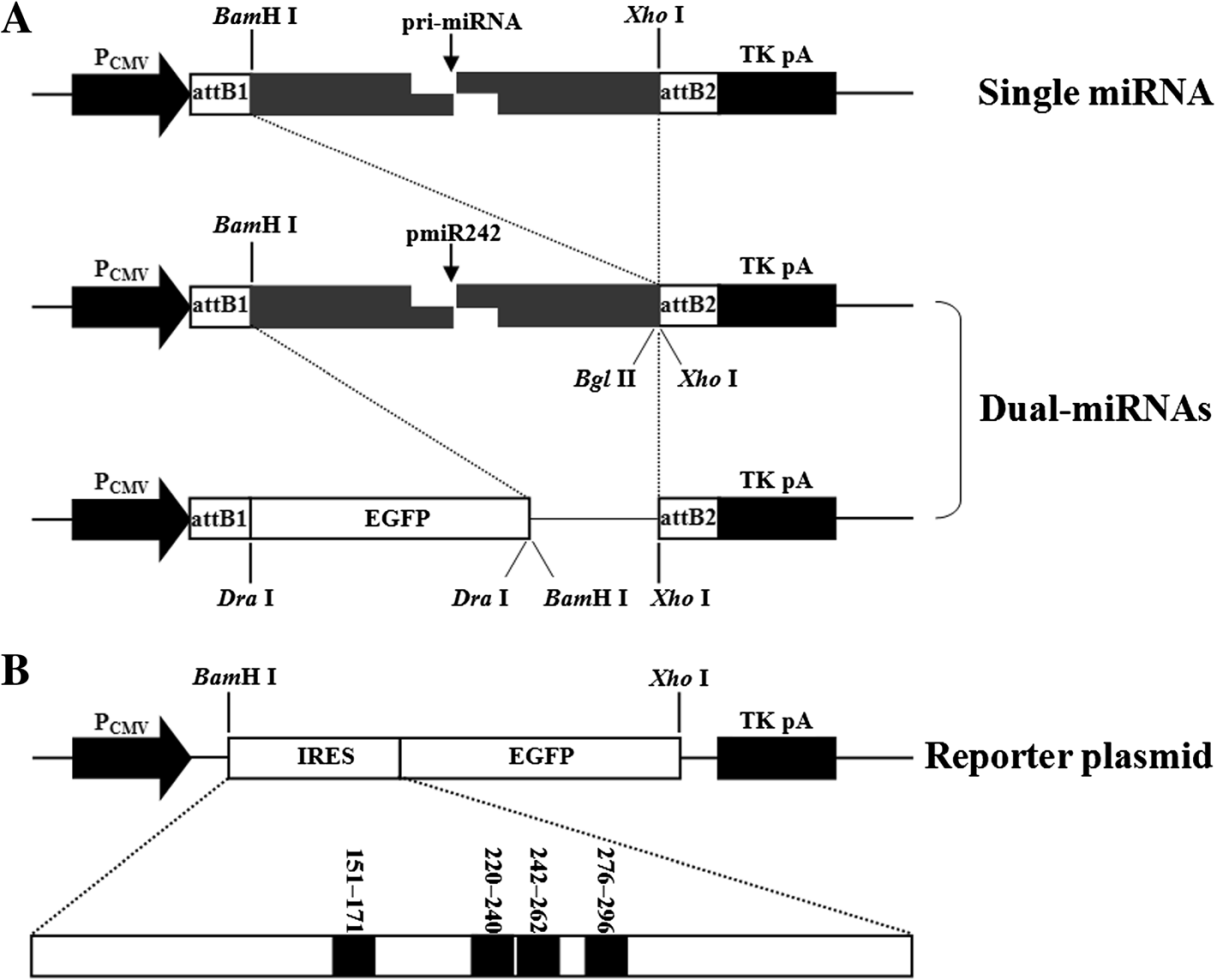
**Schematic representations of (A) FMDV-specific miRNAs and (B) IRES-EGFP expression plasmids.** 5′ and 3′ miR flanking regions are represented as grey. Procedures for the construction of single miRNA (pmiR153, pmiR220, pmiR242, and pmiR276), Dual-miRNAs (pmiR242 + 276 and pEGFP-miR242 + 276) and the reporter plasmids (pHN/IRES-EGFP, pFC/IRES-EGFP, and pJS/IRES-EGFP) are described in Methods.

**Figure 2:**
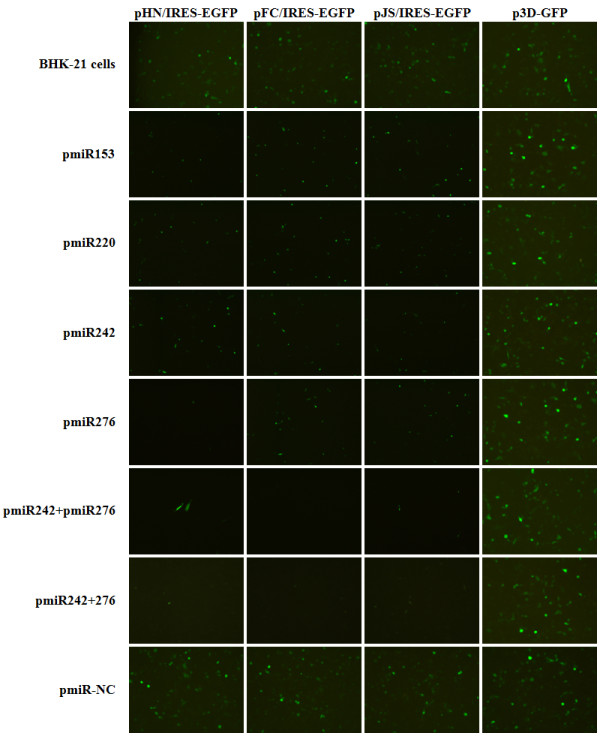
**Fluorescence micrographs of BHK-21 cells co-transfected with miRNA-expressing plasmid(s) and reporter plasmids.** The pmiR-NC and p3D-GFP plasmids were used as references for nonspecific effects on IRES-EGFP expression.

To determine if miRNAs specifically targeting the IRES element could effectively silence EGFP expression from IRES-EGFP reporter plasmids, we constructed four IRES-specific miRNA expression plasmids (pmiR153, pmiR220, pmiR242 and pmiR276; Figure 1A) and three IRES-EGFP reporter plasmids (pHN/IRES-EGFP, pFC/IRES-EGFP and pJS/IRES-EGFP; Figure 1B). Each miRNA expression plasmid (including pmiR-NC, the negative control; Table 1) was, with each reporter plasmid (including p3D-GFP, a control for nonspecific effects [41]), used to co-transfected BHK-21 cells at a molar ratio of 1:1 (w/w). The cells were observed continuously under the fluorescence microscope and analyzed by flow cytometry 48 h post-transfection. Four IRES-specific miRNAs significantly reduced EGFP expression from the IRES-EGFP reporter plasmids but not from the p3D-GFP plasmid (Figure 2). The pmiR-NC plasmid showed no visible changes in EGFP expression (Figure 2). pmiR242 (except with pHN/IRES-EGFP) and pmiR276 yielded more significant reductions of IRES-EGFP expression, compared with pmiR153 and pmiR220 (Table 2).

**Table 1:** Oligonucleotides of vector-delivered pre-miRNAs

**Name**	**Single stranded DNA sequences (5′ → 3′)**		**Position**
pmiR153	Top strand	TGCTGCTCCTTGGTAACAAGGACCCAGTTTTGGCCACTGACTGACTGGGTCCTTTACCAAGGAG	151–171
	Bottom strand	CCTGCTCCTTGGTAAAGGACCCAGTCAGTCAGTGGCCAAAAC ***TGGGTCCTTGTTACCAAGGAG*** C	
pmiR220	Top strand	TGCTGGCACGGCAACTTTACTGTGAAGTTTTGGCCACTGACTGACTTCACAGTAGTTGCCGTGC	220–240
	Bottom strand	CCTGGCACGGCAACTACTGTGAAGTCAGTCAGTGGCCAAAAC ***TTCACAGTAAAGTTGCCGTGC*** C	
pmiR242	Top strand	TGCTGCCACCTTAAGGTGACACTGATGTTTTGGCCACTGACTGACATCAGTGTCCTTAAGGTGG	243–263
	Bottom strand	CCTGCCACCTTAAGGACACTGATGTCAGTCAGTGGCCAAAAC ***ATCAGTGTCACCTTAAGGTGG*** C	
pmiR276	Top strand	TGCTGCACTGGTGACAGGCTAAGGATGTTTTGGCCACTGACTGACATCCTTAGTGTCACCAGTG	277–297
	Bottom strand	CCTGCACTGGTGACACTAAGGATGTCAGTCAGTGGCCAAAAC ***ATCCTTAGCCTGTCACCAGTG*** C	
pmiR-NC	Top strand	TGCTGAAATGTACTGCGCGTGGAGACGTTTTGGCCACTGACTGACGTCTCCACGCAGTACATTT	Heterologous
	Bottom strand	CCTGAAATGTACTGCGTGGAGACGTCAGTCAGTGGCCAAAAC ***GTCTCCACGCGCAGTACATTT*** C	

The sense sequences of predicted miRNAs targeting the FMDV IRES are shown in **bold** and ***italic***.

**Table 2:** Efficiencies of miRNAs targeting the FMDV IRES in inhibiting EGFP expression in BHK-21 cells as assayed by flow cytometry

**Reporter plasmid**	**Inhibition efficiency of each miRNA (%)**					
	**pmiR153**	**pmiR220**	**pmiR242**	**pmiR276**	**Bi-miRNA**	**Dual-miRNA**
pHN/IRES-EGFP	72.2%	56.7%	44.3%	81.4%	84.7%	95.0%
pFC/IRES-EGFP	38.7%	47.9%	71.4%	60.5%	88.3%	96.6%
pJS/IRES-EGFP	64.6%	37.5%	68.8%	62.5%	78.4%	83.6%

Bi-miRNA, a mixture of pmiR242 and pmiR276 plasmids; Dual-miRNA, a co-cistronic expression plasmid (pmiR242 + 276) containing two pre-miRNA (pmiR242 and pmiR276) hairpin structures. Percentage inhibition in each co-transfected (vector-delivered miRNAs and the reporter plasmids) cell population was calculated by comparison with the control cells transfected only with the same reporter plasmid at 48 h post-transfection.

#### Enhanced silencing of EGFP expression by Bi-miRNA and Dual-miRNA

pmiR242 and pmiR276 were used for further analysis of effective inhibition of IRES-EGFP reporter expression in BHK-21 cells. Co-transfection of a mixture of these two IRES-specific miRNA expression plasmids (pmiR242 and pmiR276, Bi-miRNA) with any of the three IRES-EGFP reporter plasmids resulted in a 78.4%–88.3% reduction in intensity of EGFP fluorescence, as compared with the individual plasmids of pmiR242 (44.3%–71.4%) and pmiR276 (60.5%–81.4%) (Figure 2, Table 2).

To further improve the specific silencing, we constructed pmiR242 + 276 (Dual-miRNA), a co-cistronic expression plasmid containing two IRES-specific miRNA hairpin structures (Figure 1A). BHK-21 cells were co-transfected with the Dual-miRNA plasmid pmiR242 + 276 and each IRES-EGFP reporter plasmid. Remarkably, the results showed that pmiR242 + 276 was more effective than pmiR153, pmiR220, pmiR242, pmiR276, or Bi-miRNA, and displayed 83.6%–96.6% inhibition of EGFP expression at 48 post-transfection (Figure 2, Table 2).

### Stable expression of IRES-specific Dual-miRNA confers effective inhibition of FMDV replication

#### Selection of stably FMDV-IRES-specific Dual-miRNA-transformed BHK-21 cells and goat fibroblasts

**Figure 3:**
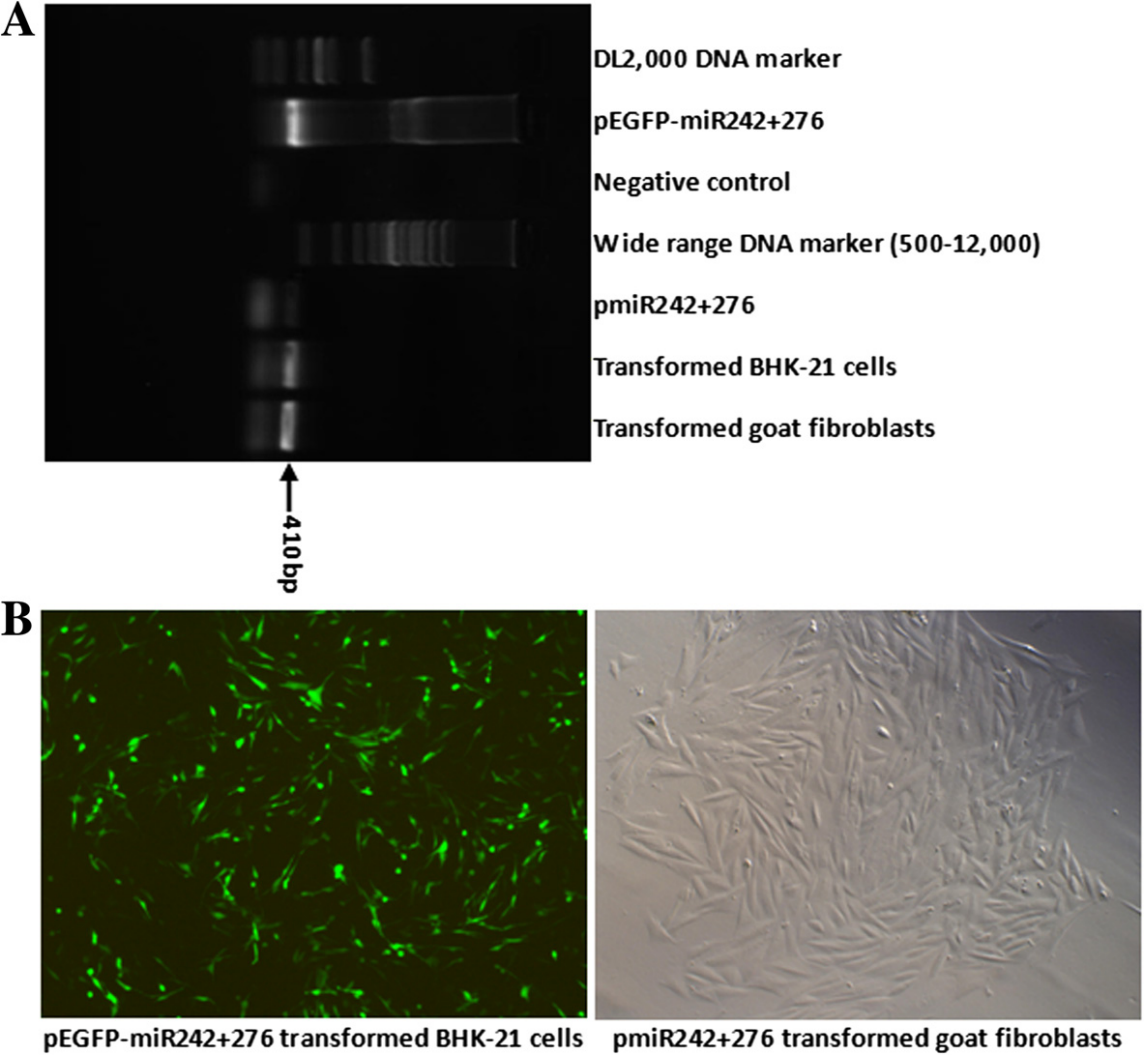
**PCR analysis and morphological observation of the FMDV IRES-specific Dual-miRNA-transformed cell clones.** Cultured cells were transfected with pEGFP-miR242 + 276 (BHK-21 cells) or pmiR242 + 276 (goat fibroblasts), and the resultant stably transformed clones were obtained by Blasticidin^R^ selection as described in Methods. **(A)** The PCR products from harvested cells were electrophoresed through 0.8% agarose gels and identified under UV light. As a negative control, a mixture of the total DNA extracted from normal BHK-21 cells and goat fibroblasts was amplified using the same specific primer pairs. **(B)** The green fluorescence in the pEGFP-miR242 + 276-transformed BHK-21 cells (left) and the pmiR242 + 276-transformed goat fibroblasts (right) were visualized with a fluorescence microscope and a light microscope, respectively.

BHK-21 cells transfected with pEGFP-miR242 + 276 (Figure 1A) and goat fibroblasts transfected with pmiR242 + 276 were grown under Blasticidin (3 μg/mL) selection for 7 days. The populations of Blasticidin-resistant (Blasticidin^R^) clones were continuously obtained in selective culture at serial passages. To determine the presence of Dual-miRNA in the transformed BHK-21 cells (the 6th passage) and goat fibroblasts (the 3rd passage), the clones were analyzed by PCR using a specific forward primer (5′-AGCAGGCTTTAAAGGGAGGTAGTG-3′) and reverse primer (5′-CTCTAGATCAAC CACTTTGT-3′). As expected, a 410-bp (base pairs) fragment was am-plified by using PCR from DNA extraction of cellular suspension from DNA extracted from every Blasticidin^R^ clone (Figure 3A). DNA sequencing revealed that the DNA containing the precursor miRNAs (pre-miRNAs) was stably integrated into the chromosomes of all the Blasticidin^R^ BHK-21 cells and goat fibroblasts. Green fluorescence was observed in the cytoplasm of the transformed BHK-21 cells (Figure 3B), suggesting that the Dual-miRNA (pEGFP-miR242 + 276) could be expressed and processed into individual IRES-specific miRNAs.

#### Analysis of inhibition of FMDV replication in Dual-miRNA-transformed BHK-21 cells

To examine the effect of IRES-specific miRNAs on FMDV replication, supernatants from virus-infected pEGFP-miR242 + 276-transformed BHK-21 cells were harvested at designated time points, total RNA was extracted, and FMDV copy numbers were measured by subjecting the total RNA samples to real-time quantitative RT-PCR. The results were very reproducible, based on the cycle threshold (Ct) values in duplicate measurements. Normal BHK-21 cells were infected with the same FMDVs as parallel controls. In cells infected with three vaccine strains of FMDV serotype O (O/HN/CHA/93), A (AF72), and Asia 1 (Asia 1/Jiangsu/China/2005) and the prevalent PanAsia-1 strain of FMDV serotype O (O/Tibet/China/1/99), the difference in mean Ct values between test samples and control samples showed that pEGFP-miR242 + 276 had the effect of inhibition on the replication of the indicated FMDV in the Dual-miRNA-transformed BHK-21 cells at 36 h post-infection ( h.p.i) (Table 3). However, viral RNA replication of the prevalent Mya98 strain of FMDV serotype O (O/CHN/Mya98/33-P) was not inhibited in the Dual-miRNA-transformed BHK-21 cells (Table 3). The results show that IRES-specific miRNAs can significantly inhibit viral infection of the selected FMDVs (except for O/CHN/Mya98/33-P) *in vitro*.

**Table 3:** Real-time quantitative RT-PCR analysis of the inhibition of FMDV replication in pEGFP-miR242 + 276-transformed BHK-21 cells, compared with normal BHK-21 cells

**FMDV**	**Ct values (mean)**					
	**12 h.p.i.**	**24 h.p.i.**	**36 h.p.i.**	**48 h.p.i.**	**60 h.p.i.**	**72 h.p.i.**
O/Tibet/China/1/99	34.61 ± 0.32/37.73 ± 0.07	36.20 ± 0.09/38.76 ± 0.25	35.98 ± 0.83/36.09 ± 0.12	37.55 ± 0.22/36.75 ± 0.44	37.62 ± 0.51/26.92 ± 0.21	36.08 ± 0.08/28.49 ± 0.39
O/HN/CHA/93	36.45 ± 0.05/35.65 ± 0.66	36.85 ± 0.57/31.32 ± 0.10	33.41 ± 0.15/28.62 ± 0.40	28.54 ± 0.10/28.82 ± 0.49	26.84 ± 0.16/26.34 ± 0.11	26.34 ± 0.30/26.54 ± 0.22
O/CHN/Mya98/33-P	16.70 ± 0.10/16.27 ± 0.23	15.17 ± 0.02/14.57 ± 0.04	11.81 ± 0.30/12.44 ± 0.14	8.43 ± 0.59/14.16 ± 0.41	7.49 ± 0.31/9.16 ± 0.04	13.68 ± 0.10/15.02 ± 0.59
AF72	30.97 ± 0.08/25.23 ± 0.17	36.23 ± 0.78/30.44 ± 0.41	36.36 ± 0.18/35.02 ± 0.79	38.45 ± 1.07/25.21 ± 0.92	36.32 ± 0.94/10.32 ± 0.71	37.13 ± 0.15/11.16 ± 0.31
Asia1/Jiangsu/China/2005	36.73 ± 0.18/34.16 ± 0.68	37.12 ± 0.11/35.65 ± 0.19	37.28 ± 0.39/35.46 ± 0.04	35.42 ± 0.09/27.39 ± 0.20	36.62 ± 0.57/17.84 ± 0.63	38.59 ± 0.77/16.68 ± 0.44

Viral supernatants were collected at 12, 24, 36, 48, 60, and 72 h.p.i., and the Ct values were derived from two parallel experiments (treatment/control).

### Antiviral activity of vector-delivered Dual-miRNA in suckling mice

**Figure 4:**
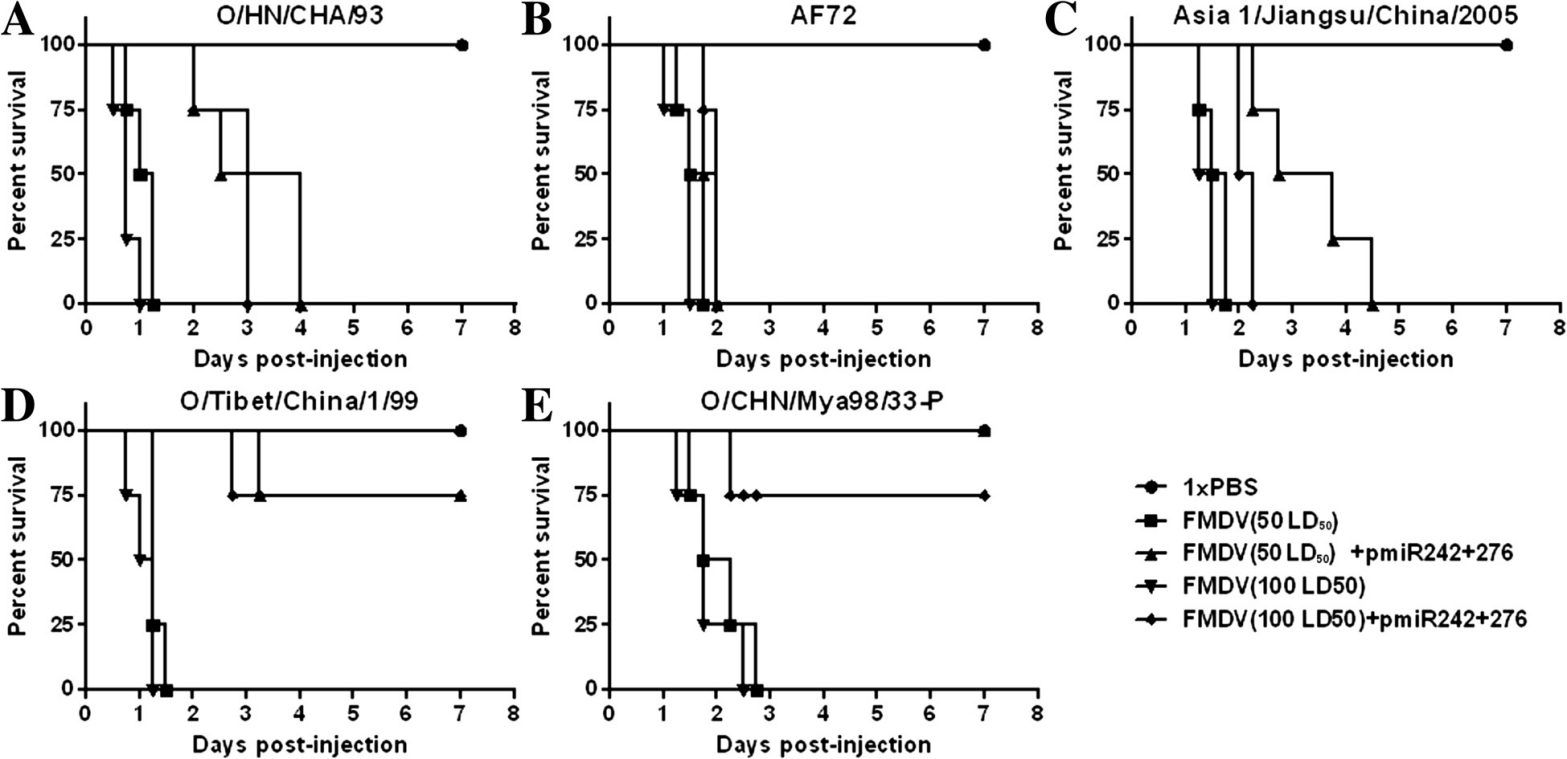
**Anti-FMDV activity of IRES-specific pmiR242 + 276 in suckling mice.** Animals (4 suckling mice of each group) were challenged by subcutaneous injection with mixtures of the miRNA expression plasmid pmiR242 + 276 and the indicated (50 LD_50_, 100 LD_50_) FMDV O/HN/CHA/93 **(A)**, AF72 **(B)**, Asia 1/Jiangsu/China/2005 **(C)**, O/Tibet/China/1/99 **(D)**, and O/CHN/Mya98/33-P **(E)**, respectively. Mice were treated with 1 × PBS as negative control and the same titers of the selected FMDVs as positive control. All suckling mice were continuously observed for one week after virus challenge. Kaplan-Meier survival curves were analyzed by the log rank test using GraphPad Prism version 5.01 (GraphPad software, San Diego, CA).

The IRES-specific miRNAs were tested against challenge by the same viruses (O/HN/CHA/93, AF72, Asia 1/Jiangsu/China/2005, O/Tibet/China/1/99, and O/CHN/Mya98/33-P) *in vivo*. All negative control mice treated with 1 × PBS survived (Figure 4). All the positive control mice, which were treated with 50 LD_50_ and 100 LD_50_ of the respective FMDVs, died within 42 h (Figure 4). In the O/HN/CHA/93, AF72, and Asia 1/Jiangsu/China/2005 challenge groups, mice that received injection of mixtures of the corresponding viruses and pmiR242 + 276 were not protected from either 50 LD_50_ or 100 LD_50_ of FMDV. However, the times at which 50% and 100% of the mice had died were delayed for more than 6 h for each of the FMDV-pmiR242 + 276-injected groups (Figure 4A, B and C). In the groups challenged with of 50 LD_50_ and 100 LD_50_ O/Tibet/China/1/99 of FMDV, 3 of the 4 mice in each group treated with mixtures of the indicated virus and pmiR242 + 276 survived for 7 days of observation (Figure 4D). Among the mice challenged with 50 LD_50_ and 100 LD_50_ O/CHN/Mya98/33-P, 4 of 4 and 3 of 4 mice, respectively, injected with mixtures of the indicated FMDV and pmiR242 + 276 also survived over the same period of time (Figure 4E).

## Discussion

miRNAs play an important role in post-transcriptional gene silencing (PTGS), inhibiting translation at and/or following initiation, as RNA interference (RNAi) [42-44]. It is believed that miRNAs are essential regulators of cell fate determination, such as in early development, and of cellular proliferation and differentiation, apoptosis, and pathogen-host interactions [45,46]. There are few reports of the use of miRNAs as anti-FMDV agents, although miRNA has more potential than siRNA to silence FMDV replication [41]. In this study, the FMDV IRES was selected as the target of vector-delivered miRNAs and the inhibitory effects of these FMDV-specific miRNAs on IRES-EGFP expression, replication of the genomic RNA, and the pathogenicity of FMDV were examined in BHK-21 cells and/or suckling mice.

**Figure 5:**
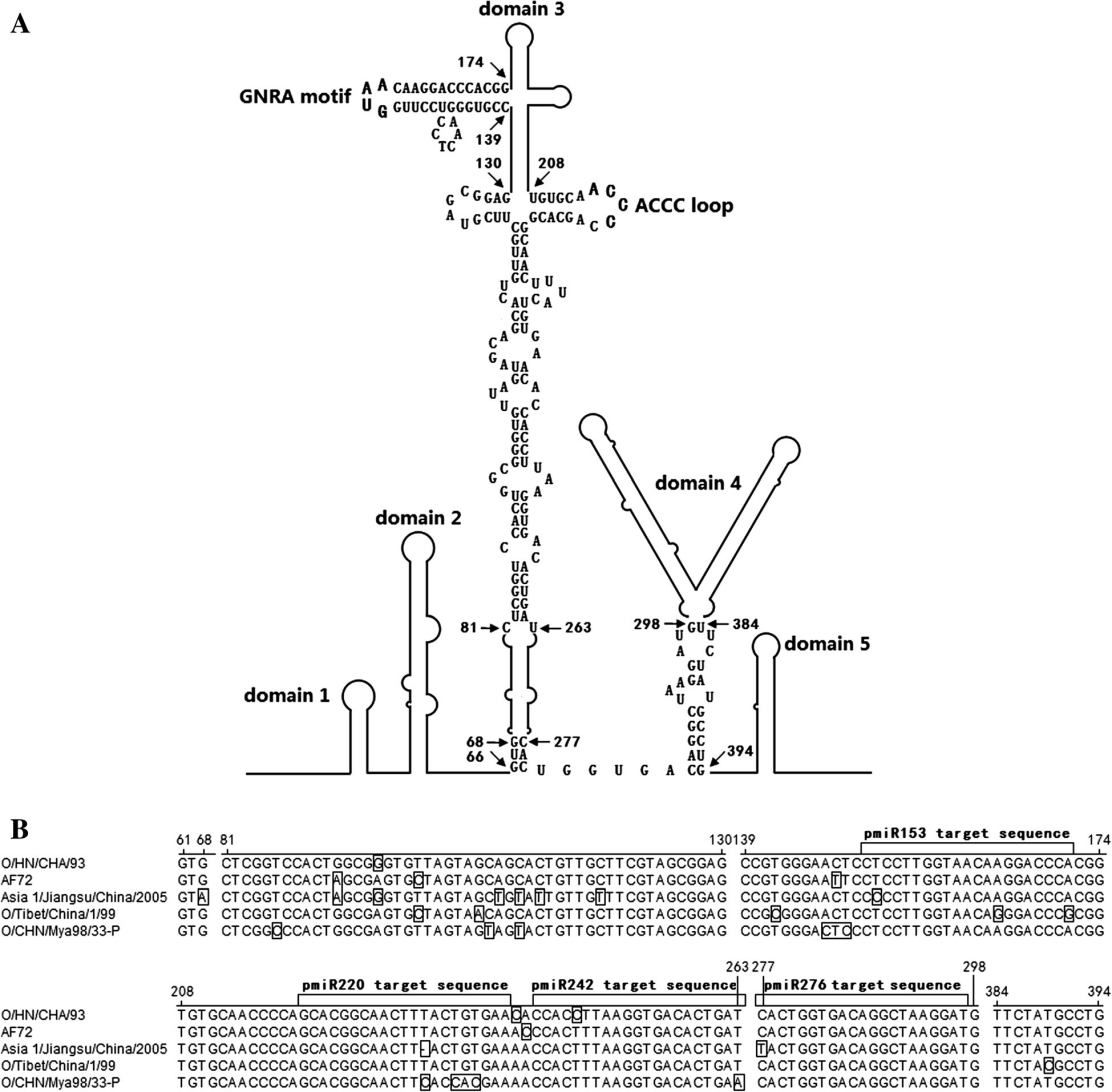
**Representation of the structural domains and locations of the miRNA target sequences within domain 3 (the central region) and domain domain 3–4 of the FMDV IRES. (A)** The secondary structure of the IRES element of FMDV O/HN/CHA/93 was predicted and referred to Kuhn et al. [11]. **(B)** The displayed sequences of the selected FMDVs were compared by using MegAlign software (DNASTAR Inc., Madison, WI) and the different nucleotides are indicated in box.

The FMDV IRES consists of a highly structured region having five structural domains [47]. The different domains have different functions in interacting with eukaryotic translation initiation factors (eIFs) to contribute to translational initiation [12,48-50]. Antisense transcripts from the 5′ region, including the proximal part of the IRES element and the functional initiator AUG codons, inhibited FMDV (serotype C) infection [51]. This inhibition, which reached values up to 90%, was dose-dependent and FMDV-specific, and also affected heterologous FMDV RNAs of serotypes O and A [52]. Rosas et al. have reported that the 156-nt transcript complementary to the FMDV translation initiation region in the viral RNA has effective antiviral activity when stably expressed in FMDV-susceptible cells [53]. In addition, small synthetic non-infectious RNA molecules corresponding to the IRES element can induce rapid, effective, and wide-range protection against FMDV infection [54]. It has been argued that the IRES element might not be accessible to RNAi [55,56]. In our experiments, the four FMDV-miRNAs targeted the GNRA motif (pmiR153), the proximal part of the ACCC loop (pmiR220) and the stem (pmiR242) of the central region (domain 3), and the root-linker-stem (pmiR276) within domain 3–4 of the IRES element, respectively (Figure 5A). Domain 3 of the FMDV IRES is unique in its ability to interact with each of the other domains, including the entire IRES [49]. The GNRA motif of the apical loops of domain 3 appears to be responsible for the organization of the adjacent stem-loops [47]. Another conserved sequence, the ACCC loop, is a candidate to interact with poly(rC) binding proteins (PCBP) [57]. Certain nts of domain 4 are involved in the interactions with proteins that play an essential role during internal initiation [58]. Here, the FMDV-specific miRNAs targeting the root-linker-stem within domain 3–4 and the stem of domain 3 provided the most efficient silencing, followed by those targeting the GNRA motif and the proximal part of the ACCC loop in domain 3 of the IRES element. The diversity of base-pair conformations in the arm of GNRA motif and the stem of domain 3 (Figure 5B) might have influenced the the inhibitory effect of pmiR153 (with pHN/IRES-EGFP and pJS/IRES-EGFP) and pmiR242 (with pHN/IRES-EGFP) on EGFP expression from the reporter plasmids (Table 2). These results suggest that conformational changes are likely to be important effectors of miRNA function and the conserved regions involving Watson-Crick base pairing within the exposed part of FMDV IRES could be potential target sequences for miRNA-induced gene silencing. We also observed that the silencing effect in BHK-21 cells could be enhanced by use of Bi-miRNA (pmiR242 and pmiR276) co-transfected with the IRES-EGFP expression reporter plasmids (Table 2). Moreover, the silencing effect of vector-delivered Dual-miRNA (pmiR242 + 276) was much stronger than either a single miRNA or Bi-miRNA (Table 2). These strategies have been used for to improve the antiviral effect, and to defend against the high genetic variability of the virus and the production of viral escape mutants [59].

To analyze the antiviral effect, all serotypes of FMDV (including vaccine strains and the prevalent strains) isolated from China, except for the SEA topotype of FMDV serotype A [60], were used to inoculate the IRES-specific Dual-miRNA-transformed BHK-21 cells and for virus challenge in suckling mice. In the suckling mice, the Dual-miRNA plasmid was delivered with the virus challenge, differing from previous studies [30,33,38,61]. Unexpectedly, the IRES-specific Dual-miRNA had no inhibitory effect on the RNA replication of FMDV O/CHN/Mya98/33-P *in vitro* despite its efficacy *in vivo* (Table 3, Figure 4E). The potential for the rapid, selective replication of the virus *in vitro* would increase the possibility of genetic changes and diversity in the populations of progeny virus (Table 3) [62,63]. Consequently, the antiviral effect was inversely proportional to the number of mismatches between the miRNA and the targeted IRES sequence, although the predicted secondary structure was tolerated (Figure 5A) [64,65]. In addition, the different gene silencing efficiencies and expression levels of the mature IRES-specific miRNAs could not guarantee complete inhibition of FMDV replication in the Dual-miRNA-transformed BHK-21 cells, and suggested that the tandem arrangement of pre-miRNAs and the reporter gene might influence the antiviral efficacy of FMDV-specific miRNA-expressing plasmids (Figure 1A) [66].

## Conclusion

Our data demonstrate that FMDV replication can be significantly inhibited by FMDV-specific miRNAs targeted to the IRES element *in vitro* and *in vivo*. Blasticidin^R^ clones of goat fibroblasts with chromosomally integrated FMDV-IRES-specific Dual-miRNA genes have also been obtained, in order to produce transgenic animals resistant to FMDV. We propose that multiple miRNAs could be effective new tools for the control of rapidly spreading FMD outbreaks in the future.

## Methods

### Cells, animals, and viruses

BHK-21 cells were grown in Dulbecco’s modified Eagle’s medium (DMEM, Gibco) supplemented with 10% fetal bovine serum (FBS, Hyclone). Goat fibroblasts were kindly provided by Prof. Baohua Ma (Northwest Agriculture & Forestry University) and cultured in DMEM/F12 nutrient mixture (Gibco) (containing 1.5 g/L sodium bicarbonate) supplemented with 10% FBS. All cell lines were incubated at 37°C with 5% CO_2_. Kunming White suckling mice, 2–3 days old and weighing 3–4 g, were purchased from Lanzhou Institute of Biological Products. Five FMDV isolates, O/Tibet/China/1/99 [PanAsia-1 strain of ME-SA (Middle East-South Asia) topotype, AF506822], O/HN/CHA/93 (vaccine strain of Cathay topotype) [67], O/CHN/Mya98/33-P [Mya98 strain of SEA (South-East Asia) topotype, JQ973889], and AF72 (vaccine strain of Asia topotype) [68], Asia 1/Jiangsu/China/2005 (vaccine strain of SEA topotype, EF149009), were preserved and provided by OIE/National Foot-and-Mouth Disease Reference Laboratory of China.

### Design and generation of vector-delivered miRNA plasmids

Four potential miRNAs were developed from the complete IRES nucleotide sequence of FMDV O/HN/CHA/93 strain by using the miRNA design tool on Invitrogen’s web site tool (http://rnaidesigner.invitrogen.com/rnaiexpress/, Table 1). Oligonucleotides of the pre-miRNAs forward and reverse strands were synthesized, annealed, and cloned into pcDNA™6.2-GW/miR vector (Invitrogen) under the control of P_CMV_ and a transcriptional termination signal (TK pA), following the manufacturer’s protocol. These plasmids were designated pmiR153, pmiR220, pmiR242, and pmiR276 (Figure 1A). For subcloning, *Bam*H I/*Xho* I digested products from pmiR276 were inserted into pmiR242 at its *Bgl* II/*Xho* I sites, resulting in pmiR242 + 276, a Dual-miRNA plasmid containing two IRES-specific miRNA hairpin structures (Figure 1A). Then, *Bam*H I/*Xho* I fragments were digested from pmiR242 + 276 and cloned into pcDNA™6.2-GW/EmGFP-miR using a BLOCK-iT™ Pol II miR RNAi Expression Vector Kit with EmGFP (Invitrogen), to generate the recombinant plasmid pEGFP-miR242 + 276 expressing EGFP (Figure 1A). The pcDNA6.2-GW/miR-negative control plasmid (pmiR-NC) was provided by Invitrogen (Table 1) and has no sequence homology with FMDV. All of these plasmids were confirmed by DNA sequencing.

### Construction of reporter plasmids

To provide a reporting system for monitoring miRNA function, three recombinant reporter plasmids pHN/IRES-EGFP, pFC/IRES-EGFP, and pJS/IRES-EGFP were constructed as follows. Briefly, IRES fragments of each FMDV of vaccine strains of serotypes A, O, and Asia 1 were obtained using RT-PCR amplification with a sense *Bam*H I-adapter primer and an antisense primer, from genomic RNAs extracted from BHK-21 cell-adapted FMDV strains (O/HN/CHA/93, AF72, and Asia 1/Jiangsu/China/2005). The EGFP sequence was amplified from the pEGFP-N1 vector (Clontech) using specific primers, and the amplification products of the FMDV-IRES fusion with EGFP were constructed by use of overlapping PCR (PrimeSTAR; TaKaRa). The PCR products were then cloned into *Bam*H I/*Xho* I-degested pcDNA™6.2-GW/miR vector (Figure 1B). The sequences of the inserts were confirmed by restriction enzyme analysis and DNA sequencing. The reporter plasmid p3D-GFP used as a control for nonspecific effects was kindly provided by Dr. Junzhen Du [41].

### Cell transfection and miRNA silencing of EGFP expression

BHK-21 cells were seeded in 6-well plates (Nunc) within 24 h before transfection. Monolayers (about 90–95% confluent) of BHK-21 cells were transiently co-transfected with 5, 10, or 20 μg of each reporter plasmid and 5, 10, or 20 μg of each miRNA expression plasmid (including a mixture of pmiR242 and pmiR276, Bi-miRNA) or pmiR-NC construct at an optimal ratio with 10 μL Lipofectamine 2000 (Invitrogen), according to the manufacturer’s instructions. The cells were examined by fluorescence microscopy (Leica) for EGFP expression at 12, 24, 36, and 48 h post-transfection.

Specific silencing of target genes to restrain EGFP expression was also examined by flow cytometry at 48 h post-transfection as follows. The co-transfected cell monolayers were dissociated with 200 μL of 0.25% trypsin after washing with 1 × PBS two times, and resuspended in a total volume of 1 mL 1 × PBS/well. After three washes with 1 × PBS, they were diluted to 1 × 10^5^–1 × 10^7^ cells/mL in 1 × PBS for analysis by FACSCalibur (Becton-Dickinson), according the manufacturer’s protocol. The EGFP fluorescence was detected by optimal excitation at 488 nm and emission at 508 nm, and the fluorescence intensity values were calculated as the percentage of the cell populations.

### Analysis of FMDV replication in Dual-miRNA-transformed BHK-21 cells

To establish BHK-21 cells stably transformed with Dual-miRNA, 10 μg pEGFP-miR242 + 276 plasmid was used to transfect 95% confluent BHK-21 cells in 35-mm plates using Lipofectamine 2000 as described above. At 4–6 h post-transfection, the OptiMEM-I (Gibco) suspended transfection complex was removed and the cells were trypsinized, diluted 10-fold, and seeded on microtitre plates (Greiner Bio-one). The cells were maintained under DMEM containing 10% FBS and 3 μg/mL Blasticidin (Invitrogen), by means of selection of resistant forms. The selection medium was changed every 2–3 days until the resultant BHK-21 cell cultures reached 100% confluency.

The stable cell monolayers were grown at a cell density of 1–2 × 10^5^/well in 6-well plates, and washed twice with 1 × PBS. Viral suspensions titrated at 30–100 plaque forming units (PFU) per 1 mL were used for virus challenge. A multiplicity of infection (MOI) of 5–50 PFU of each virus per 200 μL in DMEM was added to each well. After 1 h of adsorption, the inoculum was removed and the cells were washed twice with DMEM. Then, 2 mL of DMEM supplemented with 2% FBS and 1% antibiotic (50 μg/mL Spectinomycin, Sigma) was added to each well and the plates was incubated at 37°C with 5% CO_2_. Subsequently, supernatants were collected at designated time points, and frozen at −80°C for later real-time quantitative RT-PCR analysis as described previously [41].

### Virus challenge assay in suckling mice

To investigate the anti-FMDV activity of vector-delivered IRES-specific Dual-miRNA plasmid *in vivo*, suckling mice were used for virus challenge assay as previously described [31]. Four suckling mice in each group were treated by subcutaneous injection in the neck of mixtures of a total volume of 200 μL comprising 50 or 100 LD_50_ of each virus in 50 μL 1 × PBS mixed with 200 μg of pmiR242 + 276 plasmid in 150 μL 1 × PBS. Control mice were inoculated subcutaneously in the neck with the same titer of FMDV (positive control), or 1 × PBS (negative control). All mice were monitored every 6 hours up to 7 days.

### Establishment of FMDV-specific Dual-miRNA-transformed clones of goat fibroblasts

Goat fibroblasts were plated in 60-mm diameter dishes with 5 × 10^5^ cells in 10% FBS-containing DMEM/F12 24 h before transfection. The cells were transfected with 10 μg pmiR242 + 276 plasmid. After 4–6 h, the transfection complex was removed, and 10% FBS in DMEM/F12 with Blasticidin (3 μg/mL) was added to the cells. Cells resistant to Blasticidin were selected for one week, with medium changes about every 2–3 days. Independent Blasticidin^R^ clones were picked and expanded in the presence of Blasticidin (2 μg/mL) to avoid loss of the integrated DNAs. Cell stocks of IRES-specific Dual-miRNA-transformed clones were identified by PCR assay, and kept frozen in liquid nitrogen for further study.

## Competing interests

The authors declare that they have no competing interests.

## Authors’ contributions

YYC planned the study, constructed plasmids, and drafted the manuscript. YXD was involved in selection of the transformed cell clones. HFB performed gene-silencing experiments in BHK-21 cells. XNL, XRL, and KBM participated in the experiments on antiviral effects *in vitro* and *in vivo*. ZXL and XTL collected the field isolates and delivered background information, and XPC conceived the study. All authors reviewed and approved the final manuscript.
